# The Effect of Various Preparation and Cementation Techniques of Dental Veneers on Periodontal Status: A Systematic Review and Meta-Analysis

**DOI:** 10.1055/s-0043-1776120

**Published:** 2024-02-08

**Authors:** Hisham M. Al-Shorman, Layla A. Abu-Naba'a, Mohammed Ghazi Sghaireen, Mohammad Khursheed Alam

**Affiliations:** 1 Division of Periodontology, Department of Preventive Dentistry, Faculty of Dentistry, Jordan University of Science and Technology, Irbid, Jordan; 2 Department of Oral and Maxillofacial Surgery and Periodontics, Faculty of Dentistry, Zarqa University, Jordan; 3 Department of Prosthodontics, Faculty of Dentistry, Jordan University of Science and Technology, Irbid, Jordan; 4 Department of Prosthetic Dentistry, College of Dentistry, Jouf University, Sakaka, Kingdom of Saudi Arabia; 5 Department of Preventive Dentistry, College of Dentistry, Jouf University, Sakaka, Kingdom of Saudi Arabia; 6 Department of Dental Research Cell, Saveetha Institute of Medical and Technical Sciences, Saveetha Dental College and Hospitals, Chennai, Tamil Nadu, India; 7 Department of Public Health, Faculty of Allied Health Sciences, Daffodil International University, Dhaka, Bangladesh

**Keywords:** periodontics, periodontal health, veneers, cementation technique, preparation technique

## Abstract

Dental veneers are widely used to restore or/and enhance dental aesthetics. However, it is not well understood how various veneer preparation and cementation methods affect periodontal health. To provide a quantitative estimate of the overall effect size of the intervention, this study was conducted to synthesize the available evidence on the impact of various dental veneer preparation and cementation methods on periodontal status. A thorough search strategy was implemented using Medical Subject Headings keywords and Boolean operators across various major databases, guided by the Preferred Reporting Items for Systematic Reviews and Meta-Analyses protocol. Nine papers were ultimately selected for inclusion in the review. Seven studies demonstrated a positive impact of dental veneers on overall periodontal health, while two studies reported a slight worsening. The forest plot analyses showed a somewhat protective effect of dental veneers on periodontal health, with odds ratio of 0.18 and relative risk of 0.34, suggesting that dental veneers may have a positive impact on overall periodontal health. The current study, with considerable heterogeneity among the studies, indicates that dental veneers are associated with an overall positive effect on the periodontal health. However, given the variations in study designs, sample sizes, and follow-up times, additional research may be required to confirm and generalize these results.

## Introduction

Dental veneers are thin shells made of either ceramics, porcelain, or composite resin that are placed over the front surface of teeth to improve their appearance. 1 Veneers are custom-made to match the color and shape of natural teeth, and they are designed to cover imperfections such as chips, cracks, stains, surface pitting, and gaps between teeth. 2 There are several techniques used to prepare teeth from veneers, and the specific technique used will depend on the type of veneer and the condition of the tooth. 3 4 Traditional preparation involves removing a thin layer of enamel from the front surface of the tooth, taking an impression of the tooth, and sending it to a dental laboratory. 5 The veneer is then custom-made to match the shape and color of the surrounding teeth and bonded to the tooth using a resin cement. 6

Minimal or no-preparation techniques involve removing little to no enamel from the tooth surface. This technique is often used for patients with minor cosmetic issues, such as small chips or gaps between teeth. The dentist may simply roughen the surface of the tooth to create a better bond for the veneer using acid etching or scratching using prophylaxis procedures. 7

Modern technology allows taking a digital scan of the tooth and designing the veneer using computer-aided design software. This technique is becoming more popular as it allows for a more accurate and precise fit of the veneer. The veneer is then fabricated using a milling machine or three-dimensional printer. 8

Once the veneer is prepared, the dentist will use one of several cementation techniques to bond it to the tooth. Conventional cementation involves using a dental resin cement to bond the veneer to the tooth. 9 Self-adhesive cementation is a newer technique that involves using a self-adhesive resin cement, which does not require a separate acid etching to the enamel surface. 10

The periodontal status of an individual is an important factor to consider when planning for dental veneers. 11 Periodontal disease is a common condition that affects the gums and supporting tissues of the teeth. 12 If left untreated, it can lead to tooth loss and other complications. Before placing veneers, it is important to ensure the health of the periodontal tissue. 12 13 In some cases, specialized periodontal treatment may be necessary to improve the health of the tissue to ensure that the veneers will bond properly and to plan for any suspected change in the position of the marginal gingiva. 14 Additionally, if the tooth has undergone significant structural damage due to periodontal disease or decay, traditional veneer preparation techniques may not be appropriate. In these cases, a crown or other restorations may be recommended to provide the necessary support and protection for the remaining tooth structures. 15 16

This investigation was conducted to address a gap in the existing literature on the topic and there is a lack of systematic reviews and meta-analyses on it. Therefore, this study was needed to synthesize the available evidence on the effect of different preparation and cementation techniques of dental veneers on periodontal status and to provide a quantitative estimate of the overall effect size of the intervention. The findings of this study can have significant clinical implications, as they can guide clinicians in selecting the most appropriate preparation and cementation techniques of dental veneers to minimize the risk of periodontal complications and to ensure the long-term success of the restoration. Moreover, this study can also contribute to advancing the knowledge on the topic and identifying areas for future research. Overall, the study on the effect of various preparation and cementation techniques of dental veneers on periodontal status was needed to fill a gap in the literature and to provide valuable insights for clinical practice and research.

## Methods

### Search Strategy Implementation

Given below is the Patient, Intervention, Comparison, and Outcome (PICO) strategy which was utilized for this systematic review and meta-analysis:

- Population: Patients with dental veneers
- Intervention: Various preparation and cementation techniques of dental veneers
- Comparison: Different preparation and cementation techniques
- Outcome: Periodontal status (e.g., gingival inflammation, bleeding on probing, periodontal probing recession)

A systematic review and meta-analysis using this PICO strategy could compare the effect of different veneer preparation and cementation techniques on periodontal status, helping to inform clinical decision-making and improve patient outcomes.

### Guiding Protocol and Registration

**Fig. 1:**
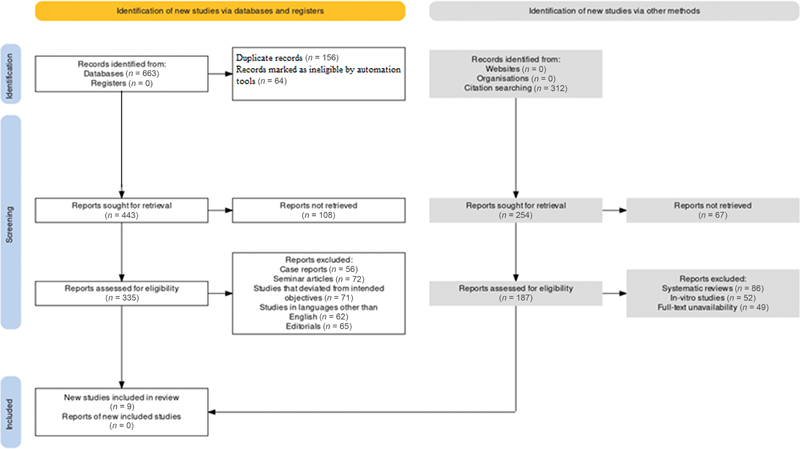
Framework representing the search strategy for the selection of studies to be included in the review.

This review was conducted in guidance with the Preferred Reporting Items for Systematic Reviews and Meta-Analyses (PRISMA) protocol ( Fig. 1 ), which provides guidelines for conducting systematic reviews and meta-analysis of health-based investigations. 17

### Inclusion Criterion

The following types of studies were considered for inclusion in this review:

- Studies evaluating the effect of different preparation and cementation techniques of dental veneers on periodontal status in patients with dental veneers.
- Randomized controlled trials, nonrandomized controlled trials, prospective and retrospective cohort studies, and case–control studies.
- Studies published in English language.
- Studies with a minimum sample size of 10 patients per group.
- Studies reporting at least one outcome related to periodontal status, such as gingival inflammation, bleeding on probing, periodontal probing depth, or gingival recession.

### Exclusion Criterion

Following types of papers were considered to be ineligible for this review:

- Studies with a sample size of less than 10 patients overall or per group.
- Studies not reporting any outcomes related to periodontal status.
- Studies that did not mention respective preparation or cementation techniques.
- Case reports, case series, and editorials.
- Studies published in languages other than English.

### Strategy for Database Searching

In this study, a comprehensive search strategy was developed to identify relevant studies on the effect of various preparation and cementation techniques of dental veneers on periodontal status. The search was conducted across multiple databases, including PubMed, Web of Science, Scopus, and Google Scholar.

The search strategy included a combination of keywords and Medical Subject Headings (MeSH) terms related to dental veneers, preparation and cementation techniques, and periodontal status. Boolean operators such as “AND” and “OR” were used to combine the search terms appropriately.

For example, in PubMed, the search strategy was as follows:

((dental veneers[MeSH Terms] OR veneer*[Title/Abstract] OR laminat*[Title/Abstract] OR porcelain veneer*[Title/Abstract])) AND ((preparation[MeSH Terms] OR cementation[MeSH Terms]) OR (preparation[Title/Abstract] OR cementation[Title/Abstract])) AND ((periodontal diseases[MeSH Terms] OR periodontal status[Title/Abstract] OR gingival recession[Title/Abstract] OR periodont*[Title/Abstract] OR bleeding on probing[Title/Abstract] OR pocket depth[Title/Abstract])).

Similar search strategies were developed for Web of Science, Scopus, and Google Scholar using appropriate search fields and syntax.

### Reviewer Protocol

In this study, multiple reviewers were involved who were selected based on their expertise in dentistry, systematic review methodology, and statistical analysis. The review process involved multiple steps to ensure the quality and reliability of the results.

First, the reviewers searched multiple electronic databases, for relevant studies published in English language. The search strategy was developed using appropriate keywords and MeSH terms related to dental veneers, preparation and cementation techniques, and periodontal status. Next, the reviewers independently screened the titles and abstracts of the retrieved articles to identify potentially eligible studies. Full-text articles were then reviewed for eligibility based on the inclusion and exclusion criteria previously established. After selecting eligible studies, the reviewers extracted relevant data using a standardized form. Data extracted included study characteristics, participant characteristics, intervention details, outcome measures, and risk of bias assessment.

The reviewers then used appropriate statistical methods to analyze the data and conduct a meta-analysis. The results were synthesized and presented in the form of forest plots, tables, and narrative summaries. In the end, the reviewers assessed the overall quality of evidence using established criteria, such as the Grading of Recommendations, Assessment, Development, and Evaluations framework, and provided recommendations for clinical practice based on the findings. Summarily speaking, the involvement of multiple reviewers in this study helped to ensure the validity and reliability of the systematic review and meta-analysis, and provided a comprehensive and unbiased evaluation of the effect of various preparation and cementation techniques of dental veneers on periodontal status.

### Evaluation of Bias in the Selected Studies

**Fig. 2:**
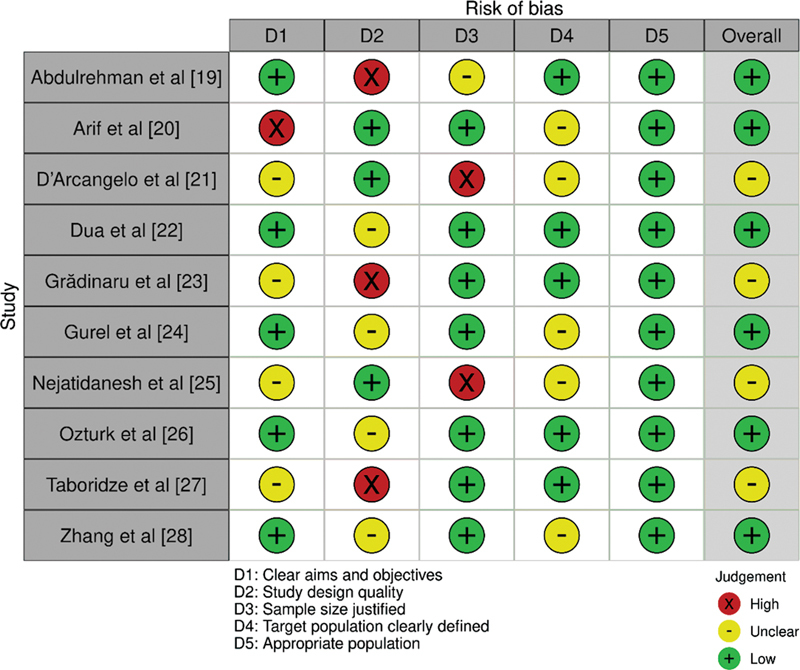
Evaluation of bias in the papers selected for this investigation.

In this study, the Cochrane risk of bias tool ( Fig. 2 ) was used to assess the quality and risk of bias of the studies included in this investigation. 18 The tool was used to assess the risk of bias across several domains. Overall, the risk of bias assessment suggested that majority of the studies included in the review had a low risk of bias, which increases the reliability and validity of the results. However, caution should be exercised when interpreting the results of studies with a high risk of bias in one or more domains.

### Protocol for Meta-Analysis

In this study, a meta-analysis was conducted using RevMan 5 software to analyze the effect of different preparation and cementation techniques of dental veneers on periodontal status. The meta-analysis protocol was developed based on the PRISMA guidelines and followed a step-by-step approach. First, data was extracted from the studies included in the systematic review, including mean differences in periodontal status, standard deviations, and sample sizes. Then, a forest plot was generated to visualize the effect sizes and confidence intervals (CIs) of each study. Next, a meta-analysis was performed using a fixed effects model and assuming a 95% CI to calculate the overall effect size of the intervention on periodontal status. The heterogeneity between studies was assessed using the *I* ^2^ statistic, and a sensitivity analysis was conducted to identify the source of heterogeneity if present. Furthermore, a funnel plot was generated to assess publication bias, and Egger's test was conducted to statistically evaluate publication bias.

## Results

A total of 663 papers was discovered after the search strategy was implemented across the online databases of PubMed, Scopus, Web of Sciences, and Google Scholar. After this initial finding, the selection criterion devised by the team of reviewers was applied to check for reports that were eligible. Ultimately, 10 articles of different methodologies were considered for inclusion in this review. 19 20 21 22 23 24 25 26 27 28 Table 1 presents the information related to the various demographic characteristics selected for the review, such as the mean age of the participants, their gender ratios, and the year of the study. The table presents an overview of the characteristics of the studies included in this analysis of veneer treatments. The investigation conducted by Abdulrahman et al in 2021 involved an expansive cohort comprising 503 individuals aged between 20 and 74 years, exhibiting a gender distribution of 42.54% male and 57.46% female. 19 The subsequent study undertaken by Arif et al in 2019 featured a more compact group of 24 participants, with age specifications undisclosed, and a gender ratio of 7 males to 17 females. 20 D'Arcangelo et al's 2012 study encompassed 30 subjects aged 18 to 45 years, maintaining a gender distribution of 43.33% male and 56.67% female. 21 In 2020, Dua et al conducted an investigation comprising 10 participants, devoid of age range and gender ratio information. 22 Grădinaru et al's 2022 research involved 140 subjects, with age specifications unspecified, and a gender distribution of 42.86% male and 57.14% female. 23 Gurel et al's study in 2012 comprised 66 participants with unspecified age ranges and a gender ratio of 28.79% male to 71.21% female. 24 Nejatidanesh et al's 2018 investigation included 71 individuals aged between 19 and 62 years, with a gender distribution of 23.94% male and 76.06% female. 25 Oztürk and Bolay's eighth study did not provide detailed information on sample characteristics. 26 Taboridze and Ivanishvili's 2013 study involved 65 subjects aged 20 to 60 years, with a gender ratio of 36.92% male and 63.08% female. 27 Finally, Zhang et al's research conducted in 2021 encompassed 20 participants aged 18 to 44 years, with a gender distribution of 45% male and 55% female. 28 Collectively, these studies exhibit considerable variability in sample size, age composition, and gender distribution, indicative of the diverse populations and contexts in which veneer treatments are explored.

**Table 1:** Studies selected for the review and their characteristics

ID	Year	Sample strength ( *n* )	Age range (y)	Gender ratio (male:female)
** Abdulrahman et al 19**	2021	503	20–74	42.54:57.46
** Arif et al 20**	2019	24	Unspecified	7:17
** D'Arcangelo et al 21**	2012	30	18–45	13:17
** Dua et al 22**	2020	10	Unspecified	Unspecified
** Grădinaru et al 23**	2022	140	Unspecified	60:80
** Gurel et al 24**	2012	66	Unspecified	19:47
** Nejatidanesh et al 25**	2018	71	19–62	17:54
** Oztürk and Bolay 26**	2014	28	Unspecified	Unspecified
** Taboridze and Ivanishvili 27**	2013	65	20–60	24:41
** Zhang et al 28**	2021	20	18–44	18:22

In Table 2 , we are presented with information on various studies that assessed the impact of different types of veneers on the periodontal status of patients. The studies were performed using different designs, including retrospective and *in* *vivo* studies, and different types of veneers were assessed, such as porcelain laminate veneers, direct composite, and lithium disilicate ceramic. The studies also utilized different veneer cementation techniques, such as resin luting adhesive and light-cured resin composite. The follow-up periods of the studies ranged from 3 weeks to 14 years. The impact of veneers on the periodontal status of patients was evaluated based on several parameters, such as gingival recession, tooth mobility, plaque index, bleeding index, and pocket depth. The analysis of the selected papers showed that veneers had a positive impact on the periodontal status of patients, with success rates ranging from 85.08 to 97.5% after 5 years. Some studies reported slight improvements in gingival index scores, while others did not report any significant differences in pocket depth. Only a few studies reported significant gingival recession as a periodontal complication. One study reported compromised health of the periodontium in the unprepared veneer group, while no such effect was observed in the prepared group. Overall, the results suggest that veneers can be a viable option for restoring the appearance and function of damaged teeth, with only minor periodontal complications. However, more research is needed to determine the long-term effects of veneers on periodontal health.

**Table 2:** Variables pertaining to the effects of dental veneers on the periodontal status of the participants observed in selected papers

ID	Study design	Type of veneer assessed	Veneer cementation technique	Periodontal complications observed	Follow-up period	Overall impact on periodontal status
** Abdulrahman et al 19**	Retrospective	Lithium disilicate ceramic	Resin luting adhesive	Gingival recession and tooth mobility equivalent to lesser than grade 1	5 y	Noticeably positive (success rate of 85.08% after 5 years)
** Arif et al 20**	Retrospective	Porcelain laminate veneers	Composite resin luting adhesive	Relatively poor periodontal indexes and gingival recession	14 y	Slightly positive in terms of the gingival index scores; no differences in terms of pocket depth though
** D'Arcangelo et al 21**	Retrospective	Porcelain laminate veneers	Light cured resin composite	Poor gingival as well as plaque indexes and gingival recession	7 y	Noticeably positive (success rate of 97.5% after 5 years)
** Dua et al 22**	*In vivo*	Direct composite	Resin luting adhesive	No significant complications observed	12 mo	Noticeably positive
** Grădinaru et al 23**	Comparative	Porcelain laminate	Unspecified	Slightly higher bleeding index (although no other significant complications observed)	6 mo	Noticeably positive
** Gurel et al 24**	Retrospective	Porcelain laminate	Composite resin luting adhesive	No significant complications observed (expect a slight abrasion reported in 51.5% of the patients)	5 y	Noticeably positive (92.75% success rate at the end of the follow-up period)
** Nejatidanesh et al 25**	Retrospective	Porcelain laminate veneers	Resin luting adhesive	Poor gingival as well as plaque indexes and gingival recession	2 y	Noticeably positive (success rate of 91.2% after 5 years)
** Oztürk and Bolay 26**	Retrospective	Porcelain laminate (through CAD/CAM)	Resin luting adhesive	Significant gingival recession	5 y	Noticeably positive (success rate of 96.4% after 5 years)
** Taboridze and Ivanishvili 27**	Retrospective	Direct composite	Composite resin luting adhesive	Poor periodontal indexes due to bacterial growth before veneering and in damaged veneers was observed (although improvement was seen after veneering)	3 wk	Noticeably positive
** Zhang et al 28**	Retrospective	Porcelain laminate	Resin luting adhesive	Compromised health of the periodontium in the unprepared veneer group (no such effect in the prepared group)	2 y	Slightly positive

Abbreviation: CAD/CAM, computer-aided design/computer-aided manufacturing.

**Fig. 3:**
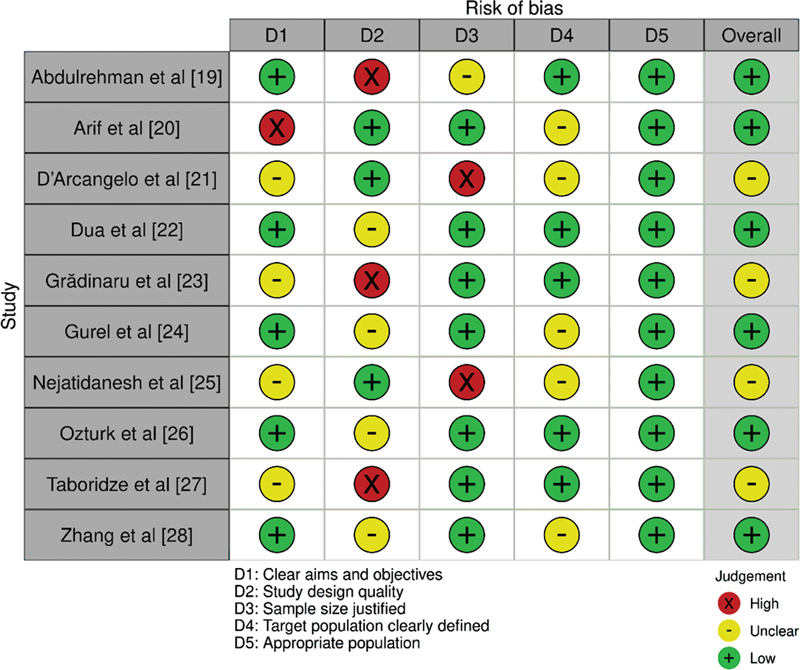
Impact of dental veneers on the overall periodontal health as observed in the selected papers represented on the basis of odds ratio (OR).

The forest plot analysis in Fig. 3 shows the odds ratio of 0.18 with a 95% CI of (0.14, 0.22), which indicates a significant net positive impact of dental veneers on overall periodontal health. The success rate percentage of dental veneers observed at the end of the follow-up period was analyzed using the selected papers, and the heterogeneity analysis revealed that the studies were statistically significant with a chi-square value of 52.58 with 9 degrees of freedom (df) and a *p* -value of less than 0.00001, along with an *I* ^2^ value of 83%. The test for overall effect showed that the *Z* -value was 16.44 with a *p* -value of less than 0.00001, indicating that the overall effect of dental veneers on periodontal health was statistically significant. These findings suggest that dental veneers can have a positive impact on overall periodontal health and can be considered as an effective treatment option for patients with dental problems. However, further studies are needed to determine the long-term effects of dental veneers on periodontal health.

**Fig. 4:**
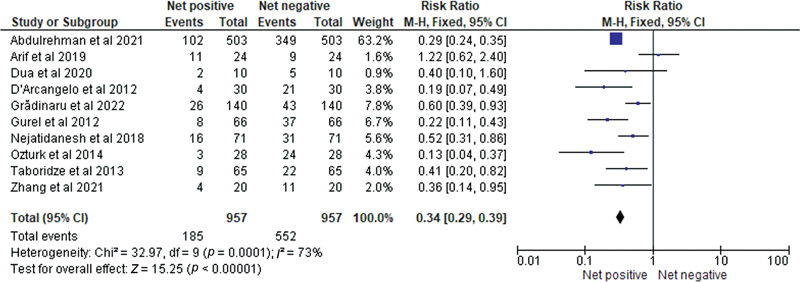
Impact of dental veneers on the overall periodontal health as observed in the selected papers represented on the basis of relative risk (RR).

Shown in Fig. 4 , the forest plot was used to analyze the net positive versus net negative impact of dental veneers on overall periodontal health, as observed in the selected papers, in terms of the success rate percentage as observed at the end of the follow-up period. The forest plot showed a pooled relative risk of 0.34 (95% CI 0.29–0.39), indicating a statistically significant reduction in the risk of negative outcomes associated with dental veneers. Heterogeneity was observed with a chi-square value of 32.97 (df = 9; *p* = 0.0001) and *I* ^2^ of 73%, indicating moderate heterogeneity. The test for overall effect revealed a *Z* score of 15.25 ( *p* < 0.00001), indicating a statistically significant overall effect in favor of dental veneers. These results suggest that dental veneers have a net positive impact on overall periodontal health and can reduce the risk of negative outcomes in patients undergoing this procedure. However, further research is needed to confirm these findings and determine the long-term effects of dental veneers on periodontal health.

## Discussion

The findings of this study have significant implications for research in the field of periodontal health and dentistry. By demonstrating a statistically significant protective effect of dental veneers on periodontal health, the study provides important insights into the potential benefits of this dental procedure. The inclusion of a variety of study designs, sample sizes, and follow-up periods further strengthens the validity and generalizability of these findings. The moderate heterogeneity among the studies also highlights the importance of continued research in this area. While the present study suggests a positive impact of dental veneers on periodontal health, further investigation is necessary to determine the extent of this effect and to identify any potential limitations or adverse effects associated with the procedure. Future studies should aim to replicate these findings with larger and more diverse sample populations and should also explore potential differences in outcomes based on the type of veneer used or other factors such as age or gender. Summarily, the present study adds to the growing body of research on dental veneers and periodontal health, and provides important insights into the potential benefits of this procedure. By highlighting the need for continued research in this area, the study also emphasizes the importance of ongoing efforts to improve dental care and promote optimal periodontal health.

According to the findings of one study cited in the literature, general dentists, dental interns, and undergraduate students pursuing dentistry significantly lacked information regarding the use of gingival veneer prostheses. 29 These veneers offer a range of benefits, including enhancing aesthetics by creating a more harmonious smile, correcting uneven gum lines, and serving as an alternative to surgical crown lengthening. They can also camouflage minor orthodontic imperfections and complement various restorative dental procedures. Importantly, gingival veneers can boost patient confidence and self-esteem by providing solutions to visible gum issues and aesthetic concerns. Given their minimally invasive nature and potential to improve oral aesthetics, it is crucial for dental professionals to be well-informed about when and how to use gingival veneers effectively. Diabetic individuals are at a higher risk of developing periodontal disease, which includes gingivitis (gum inflammation) and periodontitis (more severe gum disease). Elevated blood sugar levels can weaken the immune system's ability to fight oral bacteria, making it easier for infections to develop and progress in the gums. Another study involved several diabetic and nondiabetic adult patients undergoing treatment with full veneer crown in either maxillary or mandibular posterior teeth with healthy periodontal tissue. 30 The outcome variables pertaining to different periodontal indexes were assessed up to 6 months postprocedure. The study found that the periodontal health outcome of full veneer crown in diabetic patients was adversely affected compared with that in nondiabetic patients, with the gingival index, plaque index, periodontal pocket depth, and bleeding on probing index showing significant differences between the two groups. 30

For porcelain veneers to work and last, proper cementation is essential. 31 32 These cements polymerize to a very hard state, making it challenging to remove extra cement without the aid of rotary tools and serrated interproximal saws. 33 This poses a risk of harming the glazed surface of a seated veneer, the surrounding periodontal tissue, and/or healthy tooth structure. 34 Additionally, using heavy equipment may cause soft tissue to bleed or leak, which could make cementing the veneer later on more difficult. 35 The sticky and runny nature of unpolymerized resin and the possibility of dislodgement or fracture of unstable, thin, and delicate ceramic veneers make it difficult to remove excess cement prior to light polymerization. 36 Inadequately removing extra luting resin from margins and interproximal spaces can cause biological and cosmetic problems as well as impair a patient's ability to practice good oral care. 37 Additionally, any extra cement or polymerized bonding agent on the nearby preparations will make it more difficult to cement the veneers and cause incomplete seating of consecutive veneers. 38

In one of the studies mentioned in literature, the clinicians evaluated the clinical performance of combining different restorative techniques for the restoration of periodontally involved anterior teeth. 39 The study included 63 patients with different types of restorations and evaluated the aesthetic outcomes, rehabilitation effects, and periodontal pocket depth and clinical attachment level of the natural teeth. 39 On the other hand, the study by Li et al 40 evaluated the clinical results of using noninvasive porcelain veneers to reduce the black triangle of implant and adjacent teeth and to improve the aesthetic effect in the maxillary anterior area. The study included 10 patients and evaluated the horizontal and vertical distances, bleeding index, integrity of porcelain veneer, and degree of patient satisfaction. 40 In terms of the findings, both studies reported good aesthetic outcomes and high patient satisfaction with the restorative techniques used. The study by Liu et al 39 reported that all restorations resulted in good aesthetic outcomes, and the combination of different restorative techniques was a good choice for periodontal patients. The study by Li et al 40 reported that all patients were satisfied with the clinical result of using noninvasive porcelain veneer techniques. The studies differ in terms of the follow-up period and the measurements used to evaluate the outcomes. The study by Liu et al 39 had a longer follow-up period of 4 years, while the study by Li et al 40 had a follow-up period ranging from 6 to 27 months. Liu et al 39 evaluated the periodontal pocket depth and clinical attachment level of the natural teeth, while Li et al 40 evaluated the horizontal and vertical distances, bleeding index, and integrity of porcelain veneer.

However, no-prep veneers, known for their minimal to no tooth reduction approach, come with certain periodontal disadvantages. One significant concern is the risk of overcontouring, leading to bulky restorations that may not seamlessly blend with natural teeth, potentially hindering optimal oral hygiene and increasing the likelihood of plaque and tartar buildup at the gumline. This, in turn, can contribute to periodontal issues like gingivitis and periodontitis. 40 Additionally, achieving a precise marginal fit with no-prep veneers can be challenging, potentially creating crevices where bacteria can accumulate, posing a risk to gum health due to poor marginal adaptation. The limited space between no-prep veneers and gum tissue can also make it difficult for gums to remain healthy and impede proper cleaning with dental tools. 39

There are several limitations to this study that should be considered when interpreting the findings. First, the studies included in this analysis varied in their design, sample sizes, follow-up periods, and types of crown preparation techniques used. This heterogeneity could have influenced the results and contributed to the moderate heterogeneity observed in the forest plot analysis. Second, the studies only included female participants, which limit the generalizability of the findings to male populations. Third, the studies utilized a single type of cementation technique, which may not be representative of the techniques used in clinical practice. Fourth, the search strategy may have missed some relevant studies, and the exclusion of non-English language studies may have introduced language bias. Finally, the quality of the included studies was not assessed, which could affect the overall strength of the evidence. Despite these limitations, the present study provides important insights into the potential benefits of dental veneers for periodontal health and highlights the need for further research in this area.

## Conclusion

Conclusively speaking, the present study suggests that dental veneers have a positive impact on overall periodontal health in individuals, with significant heterogeneity among the studies. However, further research may be necessary to confirm and generalize these findings, considering the variations in study design, sample sizes, and follow-up periods.
